# Validating the cortisol hypothesis: Xanamem demonstrates positive clinical effects by lowering CNS cortisol

**DOI:** 10.1002/alz70859_102717

**Published:** 2025-12-25

**Authors:** Jack Taylor, Paul Rolan, Mark Jaros, John E Harrison, Dana Hilt

**Affiliations:** ^1^ Actinogen Medical, Sydney Australia; ^2^ Summit Analytical, Denver, CO USA; ^3^ King's College ‐ Institute of Psychiatry, Psychology & Neuroscience, London, London United Kingdom; ^4^ Alzheimer Center Amsterdam, Neurology, Vrije Universiteit Amsterdam, Amsterdam UMC location VUmc, Amsterdam, Amsterdam Netherlands; ^5^ Metis Cognition Ltd., Kilmington, Wiltshire United Kingdom

## Abstract

**Background:**

Xanamem^TM^ (Emestedastat) is an oral, selective 11β‐HSD1 inhibitor designed to reduce cortisol production in the brain under development for the treatment of AD. Clinical trials have demonstrated adequate target engagement by PET, improvements in cognitive performance in healthy older adults, and attenuation of decline in pTau181‐elevated clinically diagnosed AD patients at doses of 10mg daily. The recent positive results from the XanaCIDD trial in Major Depressive Disorder (MDD) validate the target as well as the 10mg daily dose of Xanamem.

**Method:**

XanaCIDD was a Phase 2, proof‐of‐concept trial of 10 mg Xanamem daily in MDD patients with a current depressive episode (HamD >17) and a cognitive deficit (DSST at least 0.5 SD below age and educational norms). Participants were randomized in a double‐blind design 1:1 to Xanamem 10mg or placebo for 6 weeks (Week 6), with 4 weeks follow‐up (Week 10). The primary endpoint was a customized Cogstate test battery (CTB) comprising three tests of attention and working memory at Week 6. Secondary endpoints included assessment of depression with the MADRS and Participant Global Impression of Severity scores (PGI‐S). Clinical effect sizes were described by the Cohen’s d statistic (Cd) with ≥0.2 considered to be clinically meaningful.

**Result:**

Both active and placebo groups improved substantially on the Cogstate CTB slightly favoring placebo (p=0.17). In the mITT population (n=165), a clinically and statistically significant benefit on MADRS was seen at Week 10 (2.7 points, Cd=0.43, p<0.05). In 76 patients taking SSRI medication, Xanamem benefit on MADRS was greater at the same timepoint (4.5 points, Cd=0.63, p=0.03). Xanamem showed a trend towards higher response rates at Week 10 (50% MADRS reduction, 34% Xanamem vs. 22% placebo, p=0.08). Trends towards improvement in PGI‐S scores favored the Xanamem group from Week 2 and were clinically significant at Weeks 4, 6 and 10 (p<0.2). There was a low incidence of treatment‐related adverse events which were generally mild or moderate and similar between treatment groups.

**Conclusion:**

These data suggest validation of the hypothesis that controlling CNS tissue cortisol production may be beneficial for the treatment of AD as well as MDD.

